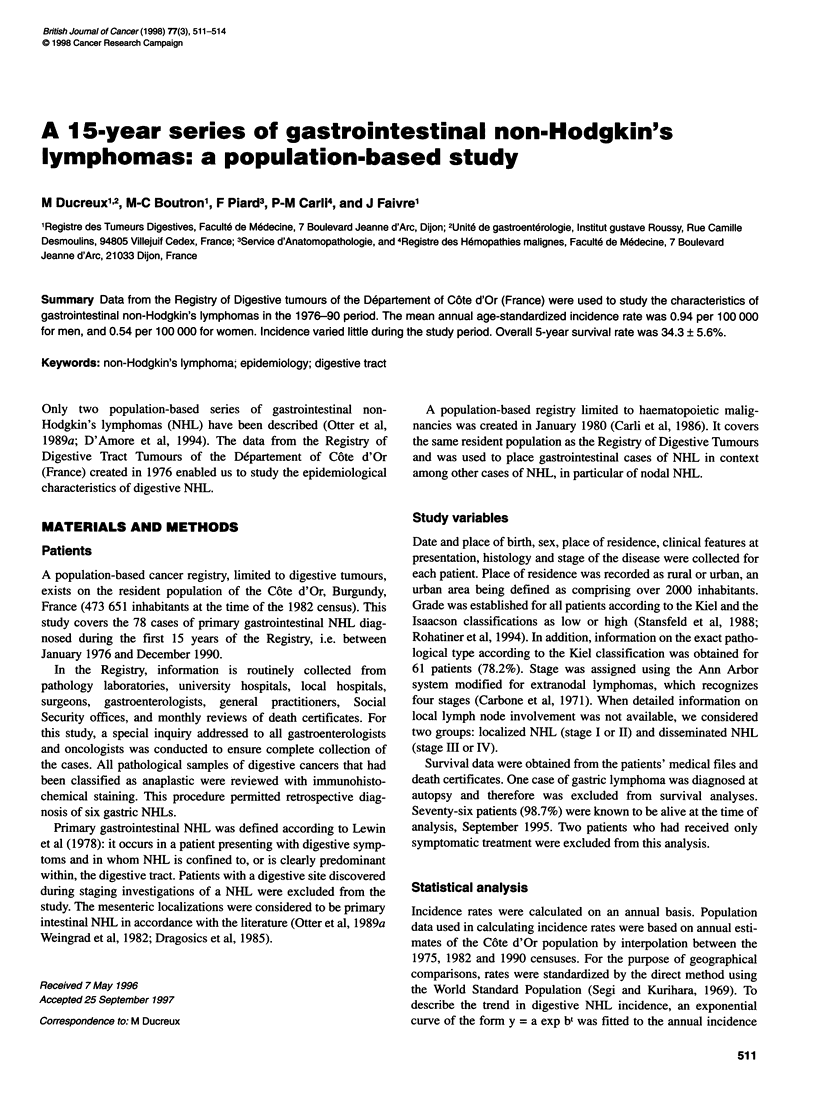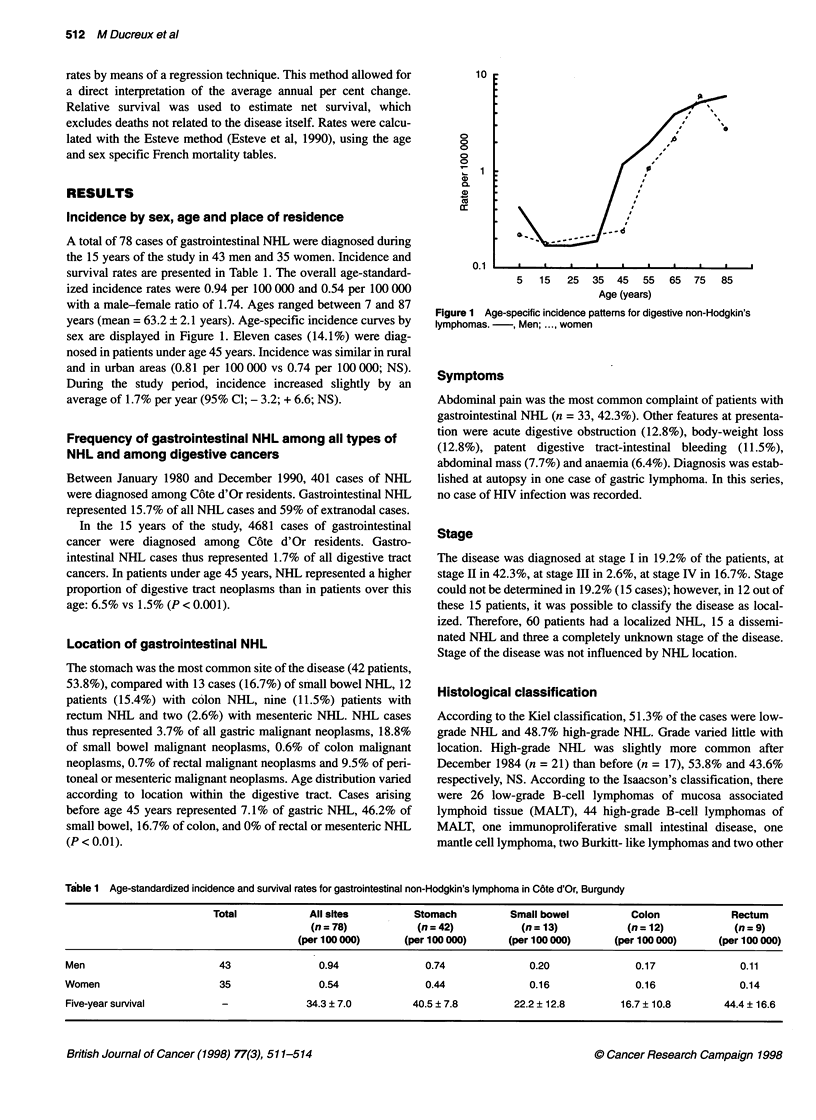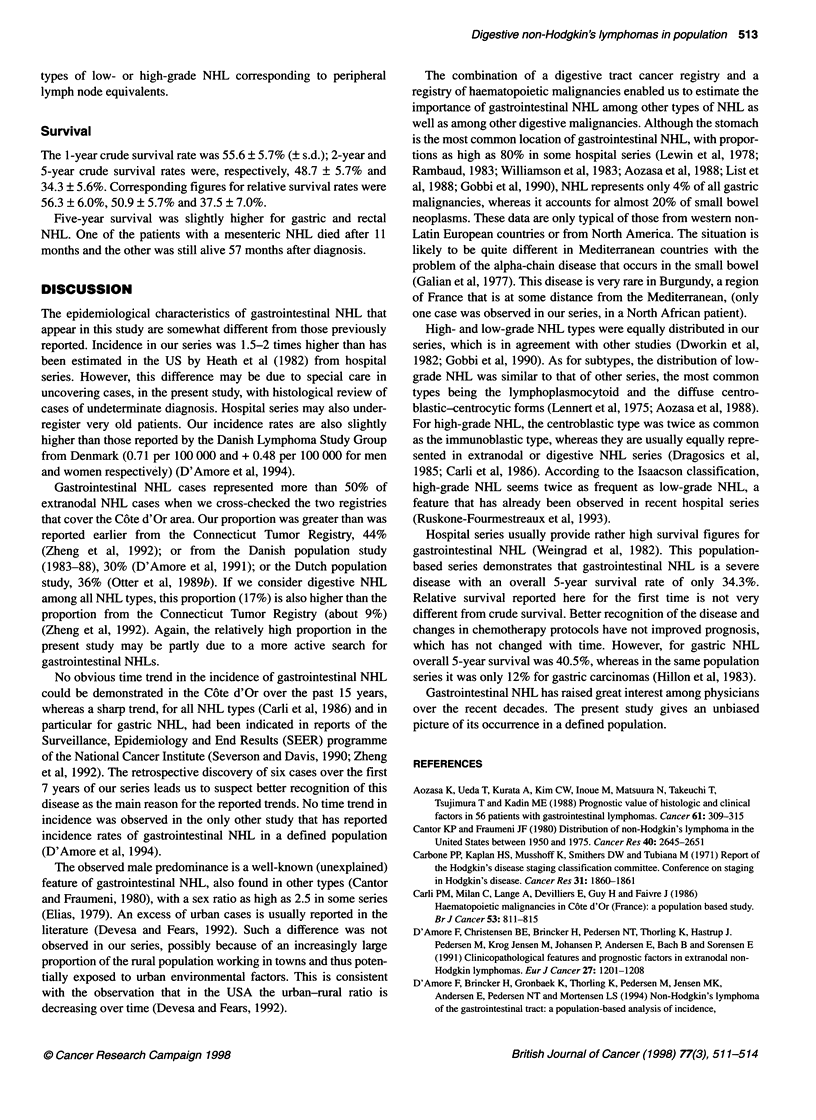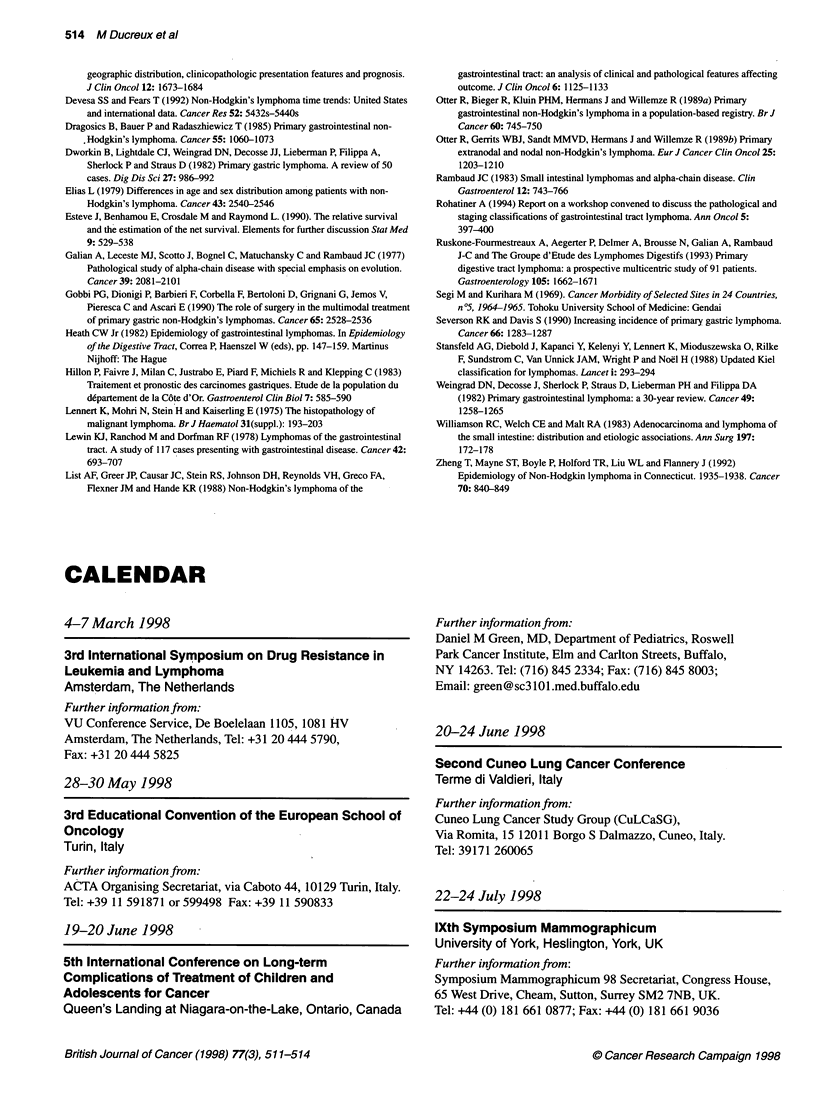# A 15-year series of gastrointestinal non-Hodgkin's lymphomas: a population-based study.

**DOI:** 10.1038/bjc.1998.82

**Published:** 1998

**Authors:** M. Ducreux, M. C. Boutron, F. Piard, P. M. Carli, J. Faivre

**Affiliations:** Registre des Tumeurs Digestives, FacultÃ© de MÃ©decine, Dijon, France.

## Abstract

Data from the Registry of Digestive tumours of the DÃ©partement of CÃ´te d'Or (France) were used to study the characteristics of gastrointestinal non-Hodgkin's lymphomas in the 1976-90 period. The mean annual age-standardized incidence rate was 0.94 per 100,000 for men, and 0.54 per 100,000 for women. Incidence varied little during the study period. Overall 5-year survival rate was 34.3 +/- 5.6%.


British Journal of Cancer (1998) 77(3), 511–514
© 1998 Cancer Research Campaign

### A 15-year series of gastrointestinal non-Hodgkin's lymphomas: a population-based study

M Ducreux[1,2], M-C Boutron[1], F Piard[3], P-M Carli[4], and J Faivre[1]

[1]Registre des Tumeurs Digestives, Faculté de Médecine, 7 Boulevard Jeanne d'Arc, Dijon; [2]Unité de gastroentérologie, Institut gustave Roussy, Rue Camille Desmoulins, 94805 Villejuif Cedex, France; [3]Service d'Anatomopathologie, and [4]Registre des Hémopathies malignes, Faculté de Médecine, 7 Boulevard Jeanne d'Arc, 21033 Dijon, France

**Summary** Data from the Registry of Digestive tumours of the Département of Côte d'Or (France) were used to study the characteristics of gastrointestinal non-Hodgkin's lymphomas in the 1976–90 period. The mean annual age-standardized incidence rate was 0.94 per 100 000 for men, and 0.54 per 100 000 for women. Incidence varied little during the study period. Overall 5-year survival rate was 34.3 ± 5.6%.

**Keywords:** non-Hodgkin's lymphoma; epidemiology; digestive tract

Only two population-based series of gastrointestinal non-Hodgkin's lymphomas (NHL) have been described (Otter et al, 1989*a*; D'Amore et al, 1994). The data from the Registry of Digestive Tract Tumours of the Département of Côte d'Or (France) created in 1976 enabled us to study the epidemiological characteristics of digestive NHL.

### MATERIALS AND METHODS

### Patients

A population-based cancer registry, limited to digestive tumours, exists on the resident population of the Côte d'Or, Burgundy, France (473 651 inhabitants at the time of the 1982 census). This study covers the 78 cases of primary gastrointestinal NHL diagnosed during the first 15 years of the Registry, i.e. between January 1976 and December 1990.

In the Registry, information is routinely collected from pathology laboratories, university hospitals, local hospitals, surgeons, gastroenterologists, general practitioners, Social Security offices, and monthly reviews of death certificates. For this study, a special inquiry addressed to all gastroenterologists and oncologists was conducted to ensure complete collection of the cases. All pathological samples of digestive cancers that had been classified as anaplastic were reviewed with immunohistochemical staining. This procedure permitted retrospective diagnosis of six gastric NHLs.

Primary gastrointestinal NHL was defined according to Lewin et al (1978): it occurs in a patient presenting with digestive symptoms and in whom NHL is confined to, or is clearly predominant within, the digestive tract. Patients with a digestive site discovered during staging investigations of a NHL were excluded from the study. The mesenteric localizations were considered to be primary intestinal NHL in accordance with the literature (Otter et al, 1989*a* Weingrad et al, 1982; Dragosics et al, 1985).

*Received 7 May 1996*
*Accepted 25 September 1997*

*Correspondence to:* M Ducreux

A population-based registry limited to haematopoietic malignancies was created in January 1980 (Carli et al, 1986). It covers the same resident population as the Registry of Digestive Tumours and was used to place gastrointestinal cases of NHL in context among other cases of NHL, in particular of nodal NHL.

### Study variables

Date and place of birth, sex, place of residence, clinical features at presentation, histology and stage of the disease were collected for each patient. Place of residence was recorded as rural or urban, an urban area being defined as comprising over 2000 inhabitants. Grade was established for all patients according to the Kiel and the Isaacson classifications as low or high (Stansfeld et al, 1988; Rohatiner et al, 1994). In addition, information on the exact pathological type according to the Kiel classification was obtained for 61 patients (78.2%). Stage was assigned using the Ann Arbor system modified for extranodal lymphomas, which recognizes four stages (Carbone et al, 1971). When detailed information on local lymph node involvement was not available, we considered two groups: localized NHL (stage I or II) and disseminated NHL (stage III or IV).

Survival data were obtained from the patients' medical files and death certificates. One case of gastric lymphoma was diagnosed at autopsy and therefore was excluded from survival analyses. Seventy-six patients (98.7%) were known to be alive at the time of analysis, September 1995. Two patients who had received only symptomatic treatment were excluded from this analysis.

### Statistical analysis

Incidence rates were calculated on an annual basis. Population data used in calculating incidence rates were based on annual estimates of the Côte d'Or population by interpolation between the 1975, 1982 and 1990 censuses. For the purpose of geographical comparisons, rates were standardized by the direct method using the World Standard Population (Segi and Kurihara, 1969). To describe the trend in digestive NHL incidence, an exponential curve of the form $y = a \exp b^t$ was fitted to the annual incidence rates by means of a regression technique. This method allowed for a direct interpretation of the average annual per cent change. Relative survival was used to estimate net survival, which excludes deaths not related to the disease itself. Rates were calculated with the Esteve method (Esteve et al, 1990), using the age and sex specific French mortality tables.

### RESULTS

### Incidence by sex, age and place of residence

A total of 78 cases of gastrointestinal NHL were diagnosed during the 15 years of the study in 43 men and 35 women. Incidence and survival rates are presented in Table 1. The overall age-standardized incidence rates were 0.94 per 100 000 and 0.54 per 100 000 with a male–female ratio of 1.74. Ages ranged between 7 and 87 years (mean = 63.2 ± 2.1 years). Age-specific incidence curves by sex are displayed in Figure 1. Eleven cases (14.1%) were diagnosed in patients under age 45 years. Incidence was similar in rural and in urban areas (0.81 per 100 000 vs 0.74 per 100 000; NS). During the study period, incidence increased slightly by an average of 1.7% per year (95% Cl; – 3.2; + 6.6; NS).

### Frequency of gastrointestinal NHL among all types of NHL and among digestive cancers

Between January 1980 and December 1990, 401 cases of NHL were diagnosed among Côte d'Or residents. Gastrointestinal NHL represented 15.7% of all NHL cases and 59% of extranodal cases.

In the 15 years of the study, 4681 cases of gastrointestinal cancer were diagnosed among Côte d'Or residents. Gastrointestinal NHL cases thus represented 1.7% of all digestive tract cancers. In patients under age 45 years, NHL represented a higher proportion of digestive tract neoplasms than in patients over this age: 6.5% vs 1.5% ($P < 0.001$).

### Location of gastrointestinal NHL

The stomach was the most common site of the disease (42 patients, 53.8%), compared with 13 cases (16.7%) of small bowel NHL, 12 patients (15.4%) with colon NHL, nine (11.5%) patients with rectum NHL and two (2.6%) with mesenteric NHL. NHL cases thus represented 3.7% of all gastric malignant neoplasms, 18.8% of small bowel malignant neoplasms, 0.6% of colon malignant neoplasms, 0.7% of rectal malignant neoplasms and 9.5% of peritoneal or mesenteric malignant neoplasms. Age distribution varied according to location within the digestive tract. Cases arising before age 45 years represented 7.1% of gastric NHL, 46.2% of small bowel, 16.7% of colon, and 0% of rectal or mesenteric NHL ($P < 0.01$).

**Figure 1** Age-specific incidence patterns for digestive non-Hodgkin's lymphomas. ——, Men; ..., women

### Symptoms

Abdominal pain was the most common complaint of patients with gastrointestinal NHL ($n$ = 33, 42.3%). Other features at presentation were acute digestive obstruction (12.8%), body-weight loss (12.8%), patent digestive tract-intestinal bleeding (11.5%), abdominal mass (7.7%) and anaemia (6.4%). Diagnosis was established at autopsy in one case of gastric lymphoma. In this series, no case of HIV infection was recorded.

### Stage

The disease was diagnosed at stage I in 19.2% of the patients, at stage II in 42.3%, at stage III in 2.6%, at stage IV in 16.7%. Stage could not be determined in 19.2% (15 cases); however, in 12 out of these 15 patients, it was possible to classify the disease as localized. Therefore, 60 patients had a localized NHL, 15 a disseminated NHL and three a completely unknown stage of the disease. Stage of the disease was not influenced by NHL location.

### Histological classification

According to the Kiel classification, 51.3% of the cases were low-grade NHL and 48.7% high-grade NHL. Grade varied little with location. High-grade NHL was slightly more common after December 1984 ($n$ = 21) than before ($n$ = 17), 53.8% and 43.6% respectively, NS. According to the Isaacson's classification, there were 26 low-grade B-cell lymphomas of mucosa associated lymphoid tissue (MALT), 44 high-grade B-cell lymphomas of MALT, one immunoproliferative small intestinal disease, one mantle cell lymphoma, two Burkitt- like lymphomas and two other

**Table 1** Age-standardized incidence and survival rates for gastrointestinal non-Hodgkin's lymphoma in Côte d'Or, Burgundy

| | Total | All sites ($n$ = 78) (per 100 000) | Stomach ($n$ = 42) (per 100 000) | Small bowel ($n$ = 13) (per 100 000) | Colon ($n$ = 12) (per 100 000) | Rectum ($n$ = 9) (per 100 000) |
|---|---|---|---|---|---|---|
| Men | 43 | 0.94 | 0.74 | 0.20 | 0.17 | 0.11 |
| Women | 35 | 0.54 | 0.44 | 0.16 | 0.16 | 0.14 |
| Five-year survival | – | 34.3 ± 7.0 | 40.5 ± 7.8 | 22.2 ± 12.8 | 16.7 ± 10.8 | 44.4 ± 16.6 |

 © Cancer Research Campaign 1998

types of low- or high-grade NHL corresponding to peripheral lymph node equivalents.

### Survival

The 1-year crude survival rate was 55.6 ± 5.7% (± s.d.); 2-year and 5-year crude survival rates were, respectively, 48.7 ± 5.7% and 34.3 ± 5.6%. Corresponding figures for relative survival rates were 56.3 ± 6.0%, 50.9 ± 5.7% and 37.5 ± 7.0%.

Five-year survival was slightly higher for gastric and rectal NHL. One of the patients with a mesenteric NHL died after 11 months and the other was still alive 57 months after diagnosis.

### DISCUSSION

The epidemiological characteristics of gastrointestinal NHL that appear in this study are somewhat different from those previously reported. Incidence in our series was 1.5–2 times higher than has been estimated in the US by Heath et al (1982) from hospital series. However, this difference may be due to special care in uncovering cases, in the present study, with histological review of cases of undeterminate diagnosis. Hospital series may also under-register very old patients. Our incidence rates are also slightly higher than those reported by the Danish Lymphoma Study Group from Denmark (0.71 per 100 000 and + 0.48 per 100 000 for men and women respectively) (D'Amore et al, 1994).

Gastrointestinal NHL cases represented more than 50% of extranodal NHL cases when we cross-checked the two registries that cover the Côte d'Or area. Our proportion was greater than was reported earlier from the Connecticut Tumor Registry, 44% (Zheng et al, 1992); or from the Danish population study (1983–88), 30% (D'Amore et al, 1991); or the Dutch population study, 36% (Otter et al, 1989*b*). If we consider digestive NHL among all NHL types, this proportion (17%) is also higher than the proportion from the Connecticut Tumor Registry (about 9%) (Zheng et al, 1992). Again, the relatively high proportion in the present study may be partly due to a more active search for gastrointestinal NHLs.

No obvious time trend in the incidence of gastrointestinal NHL could be demonstrated in the Côte d'Or over the past 15 years, whereas a sharp trend, for all NHL types (Carli et al, 1986) and in particular for gastric NHL, had been indicated in reports of the Surveillance, Epidemiology and End Results (SEER) programme of the National Cancer Institute (Severson and Davis, 1990; Zheng et al, 1992). The retrospective discovery of six cases over the first 7 years of our series leads us to suspect better recognition of this disease as the main reason for the reported trends. No time trend in incidence was observed in the only other study that has reported incidence rates of gastrointestinal NHL in a defined population (D'Amore et al, 1994).

The observed male predominance is a well-known (unexplained) feature of gastrointestinal NHL, also found in other types (Cantor and Fraumeni, 1980), with a sex ratio as high as 2.5 in some series (Elias, 1979). An excess of urban cases is usually reported in the literature (Devesa and Fears, 1992). Such a difference was not observed in our series, possibly because of an increasingly large proportion of the rural population working in towns and thus potentially exposed to urban environmental factors. This is consistent with the observation that in the USA the urban–rural ratio is decreasing over time (Devesa and Fears, 1992).

The combination of a digestive tract cancer registry and a registry of haematopoietic malignancies enabled us to estimate the importance of gastrointestinal NHL among other types of NHL as well as among other digestive malignancies. Although the stomach is the most common location of gastrointestinal NHL, with proportions as high as 80% in some hospital series (Lewin et al, 1978; Rambaud, 1983; Williamson et al, 1983; Aozasa et al, 1988; List et al, 1988; Gobbi et al, 1990), NHL represents only 4% of all gastric malignancies, whereas it accounts for almost 20% of small bowel neoplasms. These data are only typical of those from western non-Latin European countries or from North America. The situation is likely to be quite different in Mediterranean countries with the problem of the alpha-chain disease that occurs in the small bowel (Galian et al, 1977). This disease is very rare in Burgundy, a region of France that is at some distance from the Mediterranean, (only one case was observed in our series, in a North African patient).

High- and low-grade NHL types were equally distributed in our series, which is in agreement with other studies (Dworkin et al, 1982; Gobbi et al, 1990). As for subtypes, the distribution of low-grade NHL was similar to that of other series, the most common types being the lymphoplasmocytoid and the diffuse centroblastic–centrocytic forms (Lennert et al, 1975; Aozasa et al, 1988). For high-grade NHL, the centroblastic type was twice as common as the immunoblastic type, whereas they are usually equally represented in extranodal or digestive NHL series (Dragosics et al, 1985; Carli et al, 1986). According to the Isaacson classification, high-grade NHL seems twice as frequent as low-grade NHL, a feature that has already been observed in recent hospital series (Ruskone-Fourmestreaux et al, 1993).

Hospital series usually provide rather high survival figures for gastrointestinal NHL (Weingrad et al, 1982). This population-based series demonstrates that gastrointestinal NHL is a severe disease with an overall 5-year survival rate of only 34.3%. Relative survival reported here for the first time is not very different from crude survival. Better recognition of the disease and changes in chemotherapy protocols have not improved prognosis, which has not changed with time. However, for gastric NHL overall 5-year survival was 40.5%, whereas in the same population series it was only 12% for gastric carcinomas (Hillon et al, 1983).

Gastrointestinal NHL has raised great interest among physicians over the recent decades. The present study gives an unbiased picture of its occurrence in a defined population.

### REFERENCES

Aozasa K, Ueda T, Kurata A, Kim CW, Inoue M, Matsuura N, Takeuchi T, Tsujimura T and Kadin ME (1988) Prognostic value of histologic and clinical factors in 56 patients with gastrointestinal lymphomas. *Cancer* **61**: 309–315

Cantor KP and Fraumeni JF (1980) Distribution of non-Hodgkin's lymphoma in the United States between 1950 and 1975. *Cancer Res* **40**: 2645–2651

Carbone PP, Kaplan HS, Musshoff K, Smithers DW and Tubiana M (1971) Report of the Hodgkin's disease staging classification committee. Conference on staging in Hodgkin's disease. *Cancer Res* **31**: 1860–1861

Carli PM, Milan C, Lange A, Devilliers E, Guy H and Faivre J (1986) Haematopoietic malignancies in Côte d'Or (France): a population based study. *Br J Cancer* **53**: 811–815

D'Amore F, Christensen BE, Brincker H, Pedersen NT, Thorling K, Hastrup J. Pedersen M, Krog Jensen M, Johansen P, Andersen E, Bach B and Sorensen E (1991) Clinicopathological features and prognostic factors in extranodal non-Hodgkin lymphomas. *Eur J Cancer* **27**: 1201–1208

D'Amore F, Brincker H, Gronbaek K, Thorling K, Pedersen M, Jensen MK, Andersen E, Pedersen NT and Mortensen LS (1994) Non-Hodgkin's lymphoma of the gastrointestinal tract: a population-based analysis of incidence,

© Cancer Research Campaign 1998

geographic distribution, clinicopathologic presentation features and prognosis. *J Clin Oncol* **12:** 1673–1684

Devesa SS and Fears T (1992) Non-Hodgkin's lymphoma time trends: United States and international data. *Cancer Res* **52:** 5432s–5440s

Dragosics B, Bauer P and Radaszhiewicz T (1985) Primary gastrointestinal non-Hodgkin's lymphoma. *Cancer* **55:** 1060–1073

Dworkin B, Lightdale CJ, Weingrad DN, Decosse JJ, Lieberman P, Filippa A, Sherlock P and Straus D (1982) Primary gastric lymphoma. A review of 50 cases. *Dig Dis Sci* **27:** 986–992

Elias L (1979) Differences in age and sex distribution among patients with non-Hodgkin's lymphoma. *Cancer* **43:** 2540–2546

Esteve J, Benhamou E, Crosdale M and Raymond L. (1990). The relative survival and the estimation of the net survival. Elements for further discussion *Stat Med* **9:** 529–538

Galian A, Leceste MJ, Scotto J, Bognel C, Matuchansky C and Rambaud JC (1977) Pathological study of alpha-chain disease with special emphasis on evolution. *Cancer* **39:** 2081–2101

Gobbi PG, Dionigi P, Barbieri F, Corbella F, Bertoloni D, Grignani G, Jemos V, Pieresca C and Ascari E (1990) The role of surgery in the multimodal treatment of primary gastric non-Hodgkin's lymphomas. *Cancer* **65:** 2528–2536

Heath CW Jr (1982) Epidemiology of gastrointestinal lymphomas. In *Epidemiology of the Digestive Tract*, Correa P, Haenszel W (eds), pp. 147–159. Martinus Nijhoff: The Hague

Hillon P, Faivre J, Milan C, Justrabo E, Piard F, Michiels R and Klepping C (1983) Traitement et pronostic des carcinomes gastriques. Etude de la population du département de la Côte d'Or. *Gastroenterol Clin Biol* **7:** 585–590

Lennert K, Mohri N, Stein H and Kaiserling E (1975) The histopathology of malignant lymphoma. *Br J Haematol* **31**(suppl.): 193–203

Lewin KJ, Ranchod M and Dorfman RF (1978) Lymphomas of the gastrointestinal tract. A study of 117 cases presenting with gastrointestinal disease. *Cancer* **42:** 693–707

List AF, Greer JP, Causar JC, Stein RS, Johnson DH, Reynolds VH, Greco FA, Flexner JM and Hande KR (1988) Non-Hodgkin's lymphoma of the gastrointestinal tract: an analysis of clinical and pathological features affecting outcome. *J Clin Oncol* **6:** 1125–1133

Otter R, Bieger R, Kluin PHM, Hermans J and Willemze R (1989*a*) Primary gastrointestinal non-Hodgkin's lymphoma in a population-based registry. *Br J Cancer* **60:** 745–750

Otter R, Gerrits WBJ, Sandt MMVD, Hermans J and Willemze R (1989*b*) Primary extranodal and nodal non-Hodgkin's lymphoma. *Eur J Cancer Clin Oncol* **25:** 1203–1210

Rambaud JC (1983) Small intestinal lymphomas and alpha-chain disease. *Clin Gastroenterol* **12:** 743–766

Rohatiner A (1994) Report on a workshop convened to discuss the pathological and staging classifications of gastrointestinal tract lymphoma. *Ann Oncol* **5:** 397–400

Ruskone-Fourmestreaux A, Aegerter P, Delmer A, Brousse N, Galian A, Rambaud J-C and The Groupe d'Etude des Lymphomes Digestifs (1993) Primary digestive tract lymphoma: a prospective multicentric study of 91 patients. *Gastroenterology* **105:** 1662–1671

Segi M and Kurihara M (1969). *Cancer Morbidity of Selected Sites in 24 Countries, n°5, 1964–1965.* Tohoku University School of Medicine: Gendai

Severson RK and Davis S (1990) Increasing incidence of primary gastric lymphoma. *Cancer* **66:** 1283–1287

Stansfeld AG, Diebold J, Kapanci Y, Kelenyi Y, Lennert K, Mioduszewska O, Rilke F, Sundstrom C, Van Unnick JAM, Wright P and Noël H (1988) Updated Kiel classification for lymphomas. *Lancet* **i:** 293–294

Weingrad DN, Decosse J, Sherlock P, Straus D, Lieberman PH and Filippa DA (1982) Primary gastrointestinal lymphoma: a 30-year review. *Cancer* **49:** 1258–1265

Williamson RC, Welch CE and Malt RA (1983) Adenocarcinoma and lymphoma of the small intestine: distribution and etiologic associations. *Ann Surg* **197:** 172–178

Zheng T, Mayne ST, Boyle P, Holford TR, Liu WL and Flannery J (1992) Epidemiology of Non-Hodgkin lymphoma in Connecticut. 1935–1938. *Cancer* **70:** 840–849